# Employee Acceptance of Use: A Precondition for Enhancing Therapy Effectiveness, Patient Safety, and Economic Efficiency

**DOI:** 10.3389/fpubh.2019.00353

**Published:** 2019-12-05

**Authors:** Maximilian C. von Eiff, Wilfried von Eiff, Andreas Roth, Mohamed Ghanem

**Affiliations:** ^1^St. Josef Hospital Hamm (Franziskus Stiftung Muenster), Hamm, Germany; ^2^Center for Hospital Management, University of Muenster and Center for Health Care Management and Regulation, HHL Leipzig Graduate School of Management, Leipzig, Germany; ^3^Department of Orthopedics, Traumatology and Plastic Surgery, University Clinic of Leipzig, Leipzig, Germany; ^4^HHL Leipzig Graduate School of Management, Leipzig, Germany

**Keywords:** change management, innovative technology, ergonomic model, patient safety, opportunity costs, theater workflow efficiency

## Abstract

**Introduction:** From the workplace engineering sciences, it is evident that work efficiency, measured by the criteria efficiency and effectiveness of therapy, economy and patient safety, is determined mainly by staff acceptance of new technology and reengineered workflows. Accordingly, the aim of this study was to ascertain and assess differences in terms of the acceptance of alternative types of prosthesis instrument configurations, oriented around the research question: “Which product features and process effects determine a high level of employee acceptance of use?”

**Materials and Methods:** This study is designed as a before-and-after comparison, based on the usability engineering approach. In the first study phase, 46 employees participating in the process of providing instruments for a total knee arthroplasty (TKA) procedure were asked to examine the current working situation, using a standard instrumentation set, in terms of instrument handling, work burden, proneness to errors, patient risks, process efficiency, and effectiveness. In the second study phase, 20 weeks after having implemented a size-specific instrumentation set, the same 46 individuals were surveyed on the identical questions. Additionally, in both study phases the time needed to perform the sub-processes related to instrumentation logistics inside the operating room (OR) was measured, in order to identify process efficiency and cost-saving effects.

**Results:** By using standard sets only 30% perceived a need for improvement. After 20 weeks, only 8% of the employees were satisfied with the previous equipment and 69% regarded the standard set as being relatively error-prone, endangering patient safety. In addition, 85% regarded the effectiveness of the standard process as limited. Finally, 75% considered the effectiveness of the reengineered process to be significantly higher, and 69% drew attention to the reduction of handling disadvantages. Furthermore, the time needed for instrumentation logistics inside the OR estimated at about 13 min less when using size-specific sets. This effect on process efficiency cost savings or the generation of additional revenue by performing additional procedures. Based on these findings, an ergonomic decision-making model has been developed.

**Conclusion:** Innovative medical products such as size-specific instrumentation sets contribute to lower procedure costs and improved process efficiency in the operating room (OR). However, employee motivation to use a new technology as part of an optimized workflow organization, is crucial to achieving an enhanced level of effectiveness, efficiency and patient safety. Hence, it is advisable to enhance change-management efforts in order to reduce resistance to change and ensure the new technology is successful.

## Introduction

Innovative technologies, in combination with the optimization of clinical processes [e.g., introduction of surgical robots for radical prostatectomies; size-specific instrumentation sets in total knee arthroplasty (1–3)], is generally stated as the most effective way of gaining cost savings in hospitals (4) and simultaneously ensuring workplace effectiveness. We know from economic analysis and industrial ergonomic research (5) that there is a direct cause and effect correlation between a fatigue-proof work system design and productivity and quality. The same effects have also been demonstrated in various empirical studies in the field of behavioral work research [also refer to the “Hawthorne Effect” (6, 7)], as well as individual motivation theories [such as the “Dual Factor Theory” by Herzberg (8)].

Hospital research into the correlation between the design of the working environment and workplace equipment, on the one hand, and working effectiveness as well as patient risks, on the other hand, demonstrates that impairments due to stress factors from poor workplace equipment and related issues, have an adverse effect on the well-being of employees in clinical areas and lead to mistakes at work. This is the research focus of the “Healing Environment Approach” (9, 10). Empirical findings into the correlation between well-being in the workplace, on the one hand, and working efficiency and possible errors, on the other hand, are of particular importance. Distractions, which disturb ones' concentration exert a negative impact on working effectiveness and on the risk of employee errors. In this regard, various studies have examined a direct cause and effect correlation between the comfort of wearing OR (operating room) clothing and working efficiency, or the potential for errors to be made by OR personnel. For example the “Karmasin Study” (11) is based on the working hypothesis, that the comfort of surgical gowns (expressed by the quantifiable criterion “body temperature”), can influence the performance of surgeons to the extent that poor breathing of textile materials can trigger heat stress during longer surgical procedures (lasting more than 2 h), which can have an adverse impact on psychomotor characteristics (12).

A second aspect of acceptance research refers to the fact that innovative technologies are associated with changes in workflow organization and disruptions in interworking patterns. Many employees fear fulfilling the requirements of the new work environment and assume there is a risk of being burdened by additional tasks and responsibilities.

Acceptance of a new technology and the associated reorganization measures is more likely, particularly if the people affected by and those involved in a reorganization can be persuaded that:

▪ A problem exists objectively within the working process which has a considerable impact upon the situation of every individual in the workplace
▪ The current situation is associated with risks to the process (for example, patient outcome), or
▪ The overall working situation can be improved for everyone concerned by introducing a new organizational / technical concept, including with a view toward lowering costs.

Examples of the types of “improvements” which are typically requested by employees and which encourage acceptance are (13–16):

▪ Less work and reduced time pressure,
▪ Better orientation within the working process,
▪ Self-determination in clinical decision-making and independence from third-party work results,
▪ Reliability within the working process, thus making the process more fail-safe as far as the patient is concerned.

Despite these employee requirements with respect to bringing medical products with handling advantages into the working process, many hospitals prefer a price-driven procurement policy due to increasing financial pressure. Accordingly, for purchasing officers in hospitals, the price of a product is the dominant criterion when selecting and buying a medical product. Handling advantages, procedure time reduction and patient safety aspects play only a minor role in the purchasing criteria catalog. This restrictive purchasing philosophy in many hospitals is a major hurdle to bringing innovative products into practice (17).

## Goal

The overall aim of the study was to determine the importance of employee acceptance-to-work on process efficiency in the OR, tested by a planned organizational change of instrumentation sets. It was thus intended to ascertain and assess differences in terms of the acceptance of alternative types of prosthesis-instrument configurations (standard vs. size-specific instrument trays) used in total knee arthroplasty (TKA). The guiding research question was: “Which product features and process effects caused by the instrument configuration determine a high level of acceptance of use?” Furthermore, the following research hypothesis was tested and confirmed: “Employee acceptance of standard instrumentation sets is significantly lower than that of size-specific sets.”

Major objectives were to determine whether innovative medical products contribute to reducing procedure time in the OR, as well as leading to cost savings, and to determine whether handling complexity caused by medical products influences the acceptance-to-work of employees.

The study was conducted in two phases, whereby specific objectives were set for each phase.

The aim of the first study phase was to examine how satisfied employees are with the existing technical equipment, based on standard instrument sets used in a total knee arthroplasty procedure. The purpose of this first acceptance study was to establish whether and to what extent employees feel that there is a need to change the instrumentation-management process. The subsequent analysis of the organizational workflow of the instrument logistics aimed to:

▪ Identify existing medical, economic, organizational workflow or risk-related weaknesses and thus to
▪ Confirm the need to make changes to the organizational workflow.
▪ Furthermore, constructive change-management measures should be derived from these findings which help to:
▪ Accelerate learning curve effects (18, 19).
▪ Afterwards, the findings of this acceptance survey in the first study phase were ultimately factored into the specifications for the decision on the selection of new instrumentation equipment, based on size-specific surgical trays.

The aim of the second study phase was to identify differences in the acceptance behavior of employees involved in the process of instrumentation management as surgeon, scrub nurse or technician in the central sterilization department (CSSD).

## Materials and Methods

This investigation is designed as a “structural study,” a type oriented to the “Social System Approach” by Luhmann (20) and typically used in Human Relations Research represented for example by Sanders and Kianty (21). A structural study aims to identify the patterns, dynamics and stability of an organizational system (22–24) which is described by the design elements “task,” “person,” “device,” “information,” “space,” “time,” “relations” (25) and “institutions” (26). The term “institutions” refers to official rules, regulations, agreements and unwritten rules (27). Institutions are the driving factors of a socio-technical system. The productivity of a socio-technical system such as a hospital is measured by the variance in the output of this system (28).

Especially in the field of Industrial Ergonomics the “Usability Engineering Approach” of Backhaus (29), as well as the “Analytic Job Evaluation” via workload-stress-concept of Rohmert (30) are used for observational workplace research with the number of participants limited between 5 and 20. Furthermore, in lean management theory, especially in the area of patient safety through product design, standardization and work-cell design (31, 32), the generic structure of a process helps identify cause-and-effect relations as well as typical pitfalls in the process. The essence of the study is a “usability test” of an innovative size-specific instrumentation set configuration (size-specific set). Usability tests as carried out in the present study are observational studies of work systems and workflows and are quite different to the study types normally used in clinical or epidemiological studies (cohort, case control, prevalence studies). These study types are common for testing innovative procedures (e.g., TAVI: Transapical Aortic Valve Implantation) or new drugs (e.g., Multiple Sclerosis treatment regime based on Rituximab) and require more participants (*n* > 500) for ensuring statistical validity and reliability. For usability tests a minimum cohort of 5–20 procedures is recommended (33–35).

This study was conducted in a university hospital, commencing in March 2017 with the study design and the documentation of results and findings was completed in September 2018. All data related to this study excluded patient-specific data. The staff council, as the legal representative of employees in Germany (participants), consented to support the study. According to both German and European regulations, performing and publishing this study does not require ethical approval (36). Furthermore, surgical departments in Europe are free to decide which surgical instruments they use for performing a procedure, provided these instruments have been approved by a notified body (37). In this study, only instruments such approved and certified with a CE mark (Conformité Européenne) and/or conformity assessment were employed. Finally, the medical products utilized all fulfill the requirements of German (38) and European Union Law (39).

The current acceptance analysis entails a before-and-after comparison between standard sets and size-specific instrument sets. Several implants and types of sets are available on the market. In our study as well as in our practice, we use a “tibia-first” system for performing a Total Knee Arthroplasty (TKA) procedure. We start by preparing the tibial plateau and adjust further operative technical steps accordingly. Further, we performed non-navigated TKA. In all cases, the patella was not everted, the posterior cruciate ligament was retained and the collateral ligaments remained intact.

Size-specific trays only contain size-related instruments e.g., cutting guides and trial implants. These instruments are only suitable for a defined implant size, so that the surgeon has to anticipate the size of the relevant components prior to starting the procedure, based on preoperative planning. The final decision is made intraoperatively, according to the local findings and intraoperative measurement. The size of a tibial component can be measured by referring to the size of the tibial plateau after superficial bony resection. The femoral component is measured prior to bony resection. The size of the inlay always corresponds to the size of the femoral component. The thickness (height) of the inlay is measured by referring to the extension- and flexion-gap and must ensure stability as well as a sufficient range of motion. From the logistical point of view, the availability on call of different sterile trays containing possible sizes must be ensured in order to prevent any outcome risks to the patient.

In the survey, the employees involved in the sub-processes of “instrument preparation” and “cleaning up the OR,” as well as the staff responsible for cleaning and reprocessing the sets and the surgeons performing the TKA procedure, were asked via questionnaire and interviewed on the following criteria:

▪ Overall satisfaction with the respective instrumentation set in order to recognize any general need for change and to render areas of improvement transparent.
▪ Process effectivity, defined as the contribution of an instrumentation set to shorter turn-around-times, reduced risks and lower costs.
▪ Workload and time pressure in the workflow.
▪ Effectivity of quality control.
▪ Handling.
▪ Susceptibility to error.

After having delivered the completed questionnaire, all employees participating in the study were interviewed one-on-one about the reasons underlying their evaluation of the current instrumentation setting.

The survey was conducted among 46 employees (8 surgeons, 20 scrub nurses, 18 CSSD technicians) in two study phases. The first study phase (starting situation with standard set configuration) aimed at determining to what extent from the viewpoint of employees, a change in the instrumentation organization is necessary. In the second study phase carried out 20 weeks after having implemented the size-specific set type, a second acceptance analysis was accomplished in order to determine changes in employee acceptance, especially referring to “process effectiveness” and “susceptibility to error.”

In order to identify differences in process efficiency between the various instrument settings the time needed for performing the two sub-processes “preparation of all instruments” and “clearing up of used and unused instruments” was measured. “Duration” of a sub-process related to instrumentation logistics can validly be used as a proxy for process efficiency and cost savings. A reduced OR occupation time possibly allows performing an additional procedure, which yields additional revenue and contributes to avoiding higher payed overtime for the OR staff in the afternoon of a working day in the theater. Accordingly, 14 TKA procedures with standard sets and 18 TKA performed with a size-specific set were observed and the durations of the sub-processes relevant for instrumentation management were measured. Only procedures were observed in which the 46 selected employees were involved. By using this approach, confounding effects due to different learning curves and skill bases of employees, could largely be eliminated. All comparison figures and measures used are composed on Table 1.

**Table 1:** Figures, definitions, and measures used to run the comparison between two medical products and the two types of process these products characterize.

**Definitions of key figures and measures**		
**Efficiency and effectiveness of a product (here: prosthesis and instrumentation system) are measured by selected and well-defined figures**		
**Key figure**	**Definition**	**Measure**
Degree of satisfaction with a product or workflow	Product dysfunctionalities as well as working areas and workflow elements employees identify to be improved.	Questionnaire Likert scale
Perceived process effectiveness	Contribution of the process organization to shorter turn-around times, reduced risks and lower costs.	Questionnaire Likert scale
Perceived proneness to error	Probability employees perceive due to dysfunctional organization and handling disadvantages of products used.	Questionnaire Likert scale
Complexity	Number and variety of elements and interactions determining the character of a socio-technical system.	Number of instruments prepared for a procedure organized on a tray Proportion of instruments used Number of trays Floor space needed for instrumentation logistics (No. of instrumentation tables; square meters of space occupied inside OR)
Economic process effectiveness (measured via process and work analysis)	Contribution of the process organization to shorter turn-around times and lower costs.	Duration of a (sub-) process Resources needed to operate a process (working hours; salary; OR capacity)
Economic process efficiency (measured via process and work analysis)	Contribution of a process to reduced need of resources	Preparation time of instruments Cleaning up time Total process time Working time used as a proxy to determine costs and opportunity costs Overtime payment OR blockade time

## Results

The results are segmented into five areas of appraisal criteria.

(1) Employee satisfaction with current situation

**Figure 1:**
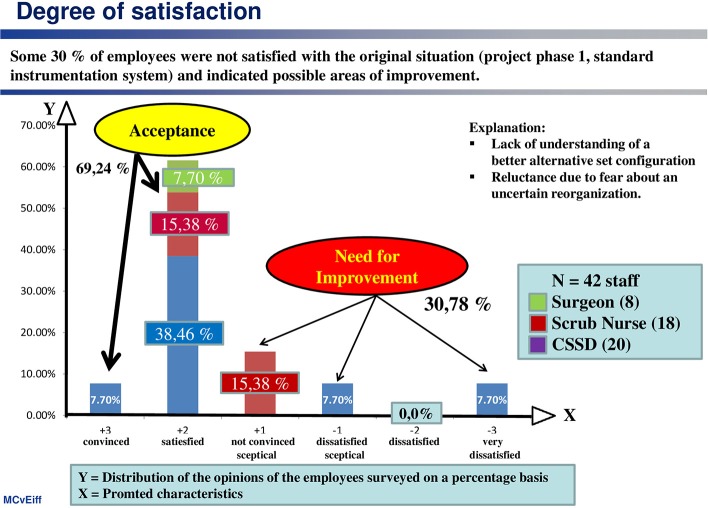
Despite a high general acceptance of the existing system, almost one third of employees felt that there was a need for improvement.

Nearly every third employee (31%) was dissatisfied with the original situation (standard instrument system) and expressed a need for change (see Figure 1). The initially high proportion (~70%) of employees who were satisfied (more precisely: “not dissatisfied”) is attributable, among other things, to two phenomena:

▪ A lack of understanding of a better alternative;
▪ Reluctance due to fears relative to an uncertain reorganization.

**Figure 2:**
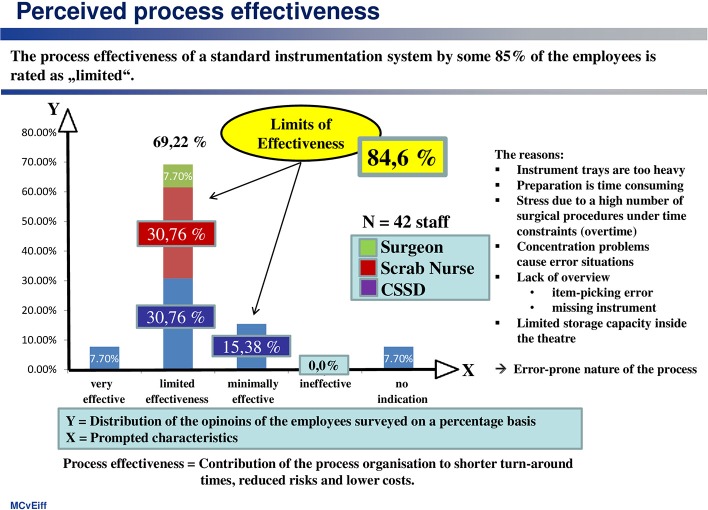
Eighty-five percentage of employees feel that the effectiveness of the process is limited.

This is illustrated by the assessment of process effectiveness (see Figure 2), which is interpreted as the “contribution made by the organization of the process (including the use of technology) to shorter instrument cycle times, reduced risk of errors and lower costs.”

According to that, only about 8% of the employees were really satisfied with the standard system. They were particularly critical of:

▪ The high weight of the trays,
▪ The time-consuming preparation process, as well as
▪ Avoidable stress resulting from a high number of surgical interventions with a limited time allowance, exacerbated by the instrument-management process.

Furthermore, the employees addressed the fact that there is a direct cause and effect relationship between the “quantity of instruments,” on the one hand, and “time pressure during the process workflow” and the “effectiveness of quality control,” on the other hand.

(2) Process effectiveness

This is defined here as the contribution of the process organization to shorter turn-around-times, reduced workflow risks for staff and patient, and top lower costs.

The employees rated the effectiveness of the standard system as “effective to a limited extent” (69.2%) or “minimally effective” (15.4%). This assessment is shared by scrub nurses and CSSD personnel alike, as well as by surgeons, albeit for other reasons. The surgeons are critical of the lack of clarity as to what is in the trays, the CSSD personnel bemoan the weight of the trays, time pressure and complexity of the process, and the scrub nurses complain about a shortage of space to accommodate the trays in the OR.

(3) Proneness to error

**Figure 3:**
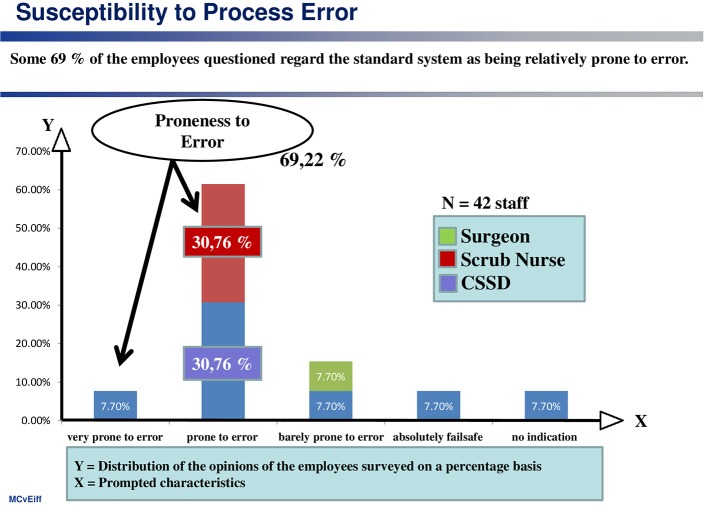
Phenomenon of being error-prone.

The proneness of the standard system to errors, due to its complexity, is also regarded as significantly high at almost 70% (see Figure 3).

(4) Complexity of handling and clarity of arrangement of instruments on trays

**Figure 4:**
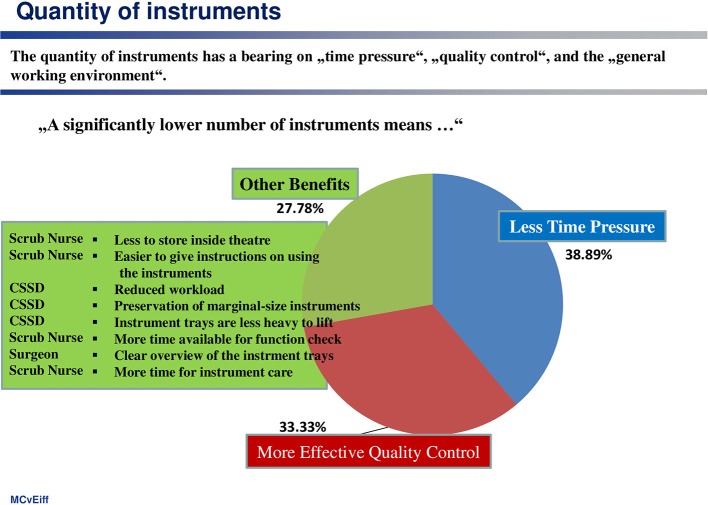
Clear organizational advantages will be achieved by reducing the number of trays and instruments.

All of the occupational groups involved feel that the overall working system would benefit greatly if there were a significant reduction in the number of trays and of instruments (see Figure 4). These advantages relate primarily to more convenient handling, greater clarity, less time pressure and a lighter workload, less error-proneness as a result of simple instructions, as well as more effective quality control.

At this point, an important cause-and-effect relation emerges: complexity and handling comfortability are experienced in terms of the weight and number of trays, which have to be used in the theater. Furthermore, the number of instruments arranged on a tray is a complexity indicator. Another complexity measure is the percentage of instruments transported back to the CSSD for reprocessing, without having been used during the TKA procedure. The before-after comparison demonstrates the use of standard instrumentation systems seems to be accompanied by a higher degree of complexity (see Table 2).

**Table 2:** The proportion of instruments placed on a tray, but not used during the procedure is a proxy indicator for work complexity and cost.

**The complexity of a prosthesis and instrumentation system is an important cost driver as well as a hidden source of failures within the scope of the instrument cycle**			
**Criteria**	**Standard setting**	**Size-specific setting**	**Reduction in complexity (%)**
Sample size	14	18	–
Number of instruments prepared	156	91	42.67%
Proportion of instruments used	32.67%	54.64%	40.21%
Number of trays	6	3	50%

As a result of the first study phase, it is evident that the personnel is generally in favor of a reduction in the number of trays and instruments.

**Figure 5:**
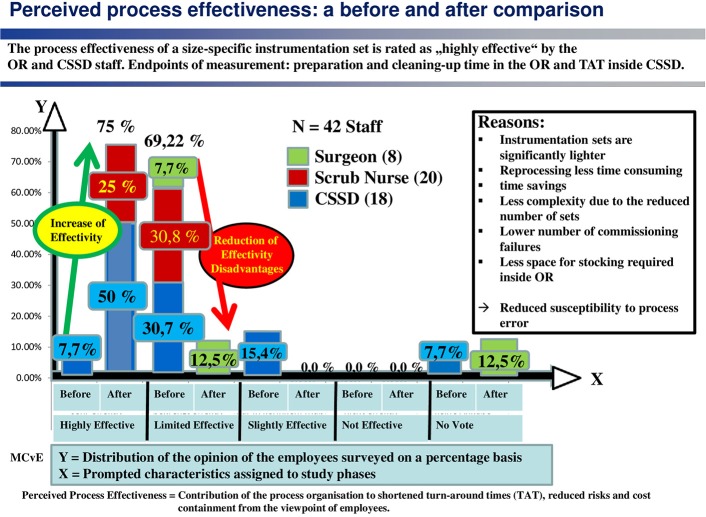
The before-and-after comparison shows a marked rise in perceived effectiveness.

Some 20 weeks after the introduction of the size-specific system, a second acceptance study was conducted in order to gauge how satisfied the personnel were with the new instrument logistics solution. The before-and-after comparison reveals a marked rise in perceived process effectiveness, i.e., in the contribution of the process organization toward shorter turnaround times, reduced risks and lower costs (see Figure 5).

Obviously, there is a very tight cause-and-effect relationship between the acceptance of an instrumentation setting and the quantity of instruments and number of trays used. Acceptance of a care variant with OR trays is essentially determined by the number of trays and the number of instruments placed in the trays. The number of instruments, which are not used, but are reprocessed, has a particularly negative effect.

**Figure 6:**
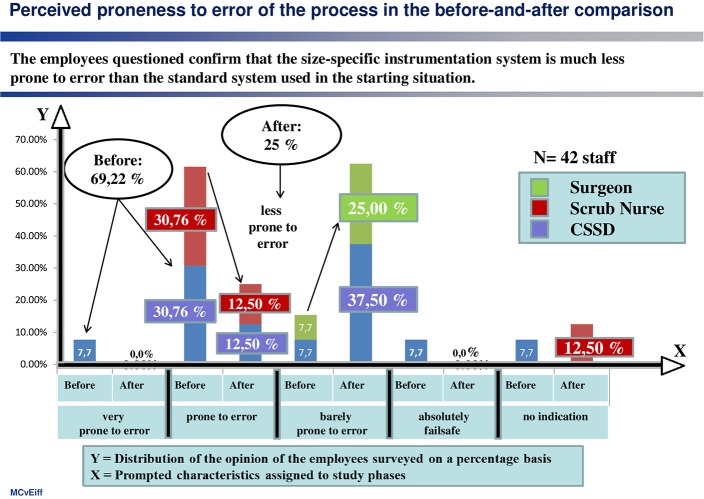
A size-specific, standardized instrument system reduces the degree to which the instrument management is error-prone.

There was also a clear improvement in satisfaction in terms of being “error-prone” (see Figure 6). For instance, 69% of those questioned regarded the standard system in the original situation as error-prone, whereas the size-specific organization was only classed as error-prone by 25%.

(5) Process efficiency

In this context process efficiency is defined as the relation between an input of resources e.g., personnel, medical products as well as devices, and the result gained by combining these resources in a well-organized medical procedure. Process efficiency is measured by the duration of uniquely defined sub-processes, the number of instruments used in relation to the number of instruments placed on a tray when starting the procedure and the direct costs as well as the opportunity costs caused by a procedure.

Opportunity costs are understood as a benefit, gain or value of something that must be given up to acquire or achieve something else (40, 41). Typical opportunity costs appear when for example an OR is blocked due to an extended procedure time as a consequence of non-availability of medical products needed for the procedure, or when a bed is occupied because a patient acquired a nosocomial infection.

**Figure 7:**
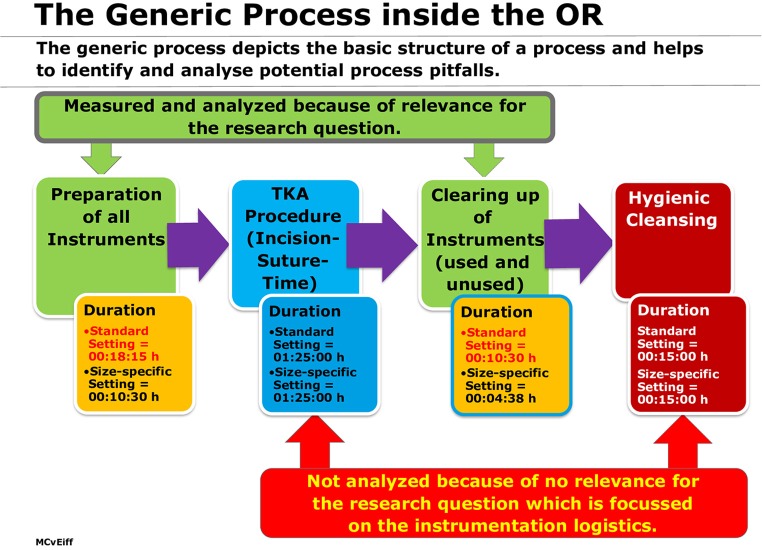
Generic process with a focus on working steps inside the OR related to instrumentation logistics.

In addition to these findings, using size-specific instrument sets contributes significantly to shortening the duration of the sub-processes for “preparing instrumentation equipment before starting operation” (incision time) and “cleaning up of used and unused instruments” after the surgeon has finished the procedure (suture time). “Process time” can be used as a proxy for process efficiency, because the time saved can be used alternatively for performing an additional procedure that yields additional revenue or for avoiding expensive overtime hours for the staff in the afternoon of a working day in the OR. In order to obtain evidence on this issue, a generic process model was constructed, which depicted the sub-processes performed in the context of a TKA procedure (see Figure 7).

The total process for the instrument handling was reduced from 28:45 to 15:08 min, which corresponds to a time gain and reduction of the occupancy time of the OR of 13:22 min (53%) for one TKA operated (see Table 3).

**Table 3:** Comparison of the durations of sub-processes related to instrumentation logistics.

**Process efficiency**			
**The duration of the sub-processes related to instrumentation logistics is significantly (53%) shorter when using size-specific instrument sets, a remarkable contribution to process efficiency and cost reduction**.			
**Sub-process in the OR**	**Standard instrumen-tation set (duration: min.)**	**Size-specifics instrumen-tation set (duration: min.)**	**Time saved (min.)**
Preparation of all Instruments	18:15	10:45	07:30
Cleaning up OR	10:30	04:38	05:52
**Total process time**	**28:45**	**15:23**	**13:22**
Opportunity cost calculation:			
If 4 Total Knee Arthroplasty procedures (TKA) were performed in one			
OR a day 53 min will be saved			
This time gained can be used for			
Avoiding overtime in the afternoon or			
Performing an another procedure achieving additional revenue			

On premise that 4 TKA are performed over one working period per day, the theater is occupied for 08:35:00 h. A time reduction of 13:22 min per TKA procedure leads to a reduced occupancy time of the OR by 53:28 min on average. This time saved can be used to perform an additional procedure, e.g., an Arthroscopy (including biopsy). The procedure time is usually 30 min, so that two efficiency effects can be identified:

▪ The total theater occupation time is reduced from 08:35 to 08:30 h.
▪ For the additional procedure (Arthroscopy) in the German DRG reimbursement system the statutory medical funds pay € 2.564 (G-DRG I24A; cost weight: 0,723; base rate: € 3.535) (42). For measuring the financial return from a surgical procedure the “Direct Costing Method” is generally used (43). In the case described, the contribution margin I (CM I = DRG reward minus variable costs of the procedure) is used to define the opportunity costs of a non-performed procedure (in this case = € 1.671) and the contribution margin II (CM II = CM I minus fixed costs associated with the procedure) defines the profit of the department before reduced by the allocatable costs of the hospital (44).

(6) Summary of results

The table of results (see Table 4) summarizes the major findings of the study and indicates the superiority of size-specific instrument trays. Moreover, it is evident that the sustainable success of innovative medical technologies in practice e.g., new products, re-organized workflows or disruptive procedures, depends on the acceptance-to-work of the employees working in the re-organized process. And this acceptance is heavily influenced by the impact of the innovative technology on procedure handling, costs, workflow efficiency, and patient safety. All these aspects are a direct result of the nature of design of an innovative technology.

**Table 4:** Results according to selected criteria.

**Table of results**			
**Criteria**	**Standard set**	**Size-specific set**	**Remarks**
**Work Complexity** No. of sets No. of Instruments prepared Floor space needed forinstrumentation logistics	6 sets 156 high	3 sets 91 low	Number of sets and instruments determine workplace complexity and influence handling and safety of the procedure
**Efficiency** OR occupation time (instrumentation-related) Instruments used (proportion)	28:45 min 32.67%	15:23 min 54.64%	Time saved is a proxy for process efficiency and cost reduction
**Process effectiveness** = contribution to shortened turn-around-times, reduced risks and cost containment	Employees judge “limited” (69%) or “minimal effective” (15%) 7.7% judge “highly effective”	12.5% judge “limited effective” 75% judge “highly effective”	Individual opinion of employees (nominal measurement by Likert scale)
**Handling advantages**	High weight of sets	Less heavy to lift the sets	Individual opinion of employees (Likert scale)
**Safety**			
Overview (instrument trays) Instructions/Learning Curve	Limited overview Complicated to give Instructions to scrub Nurses and assisstants	Clear overview Eease of giving instructions how to handle instruments	Individual opinion of employees (Likert scale)
Proneness to error quota	69% quota	25% quota	
**Opportunity costs and opportunity benefits**	Opportunity costs = 1.671€	Opportunity benefits = 53:28 min. time saving p. theater day	Time savings usable for overtime reduction or an additional procedure
**Overall aspects**	Cumbersome set handling leads to time consuming working steps and press of work	Less time pressure More effective quality Control More convenient handling	Size-specific sets contribute to a significant higher level of employee job satisfaction

## Discussion

This comparative analysis demonstrates a significant improvement in process effectiveness in terms of shortened turn-around times, reduced risks and lower costs. Also, the susceptibility to error caused by the complexity of the standard instrumentation set was originally rated significantly higher (70%).

This study also demonstrates that a lower number of instruments arranged on a tray contributes to a more stress-free and ergonomic work environment, to more effective quality control and a reduction in time pressure.

Another important finding concerns the change management organization, which is crucial for the economic success of a new implemented technology or workflow.

Moreover, it should be noted that the quality of change-management (key user support, help desk, training, tryout opportunities) contributes to shortening the learning phase and raising acceptance.

Furthermore, it is evident that the percentage of employees who class an instrument system as “error-prone” after a switchover is all the higher, the shorter the period of time between the system switchover and the acceptance study. The reason for this is that a short timeframe does not permit learning-curve effects so that personnel remain rather unsure about how to use the new and unfamiliar system.

Referring to the previously mentioned price-driven procurement philosophy, which guides many hospitals, and bearing all findings of this study in mind, it is highly advisable to change the procurement policy for purchasing medical products and devices used during surgical procedures from a price-driven management approach to a value-based philosophy.

**Figure 8:**
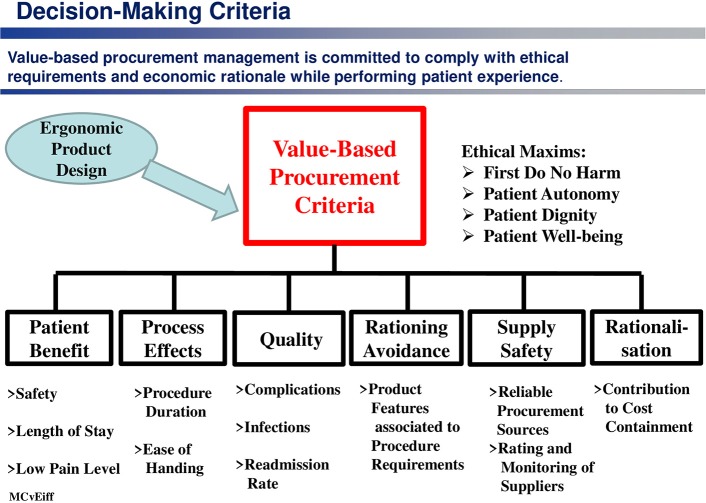
Decision-making criteria in a value-based procurement approach.

The value of a medical procedure is defined as the relation between patient outcome, patient safety, procedure time, patient pain level and patient length of stay on the one hand, and procedure costs on the other hand. Accordingly, the most important assessment criteria (45) for selecting the right medical product within the frame of a procurement decision-making process are “usability” (ease of use, avoidance of patient risks, low workload), learning-curve effects and reduced process time (see Figure 8).

**Figure 9:**
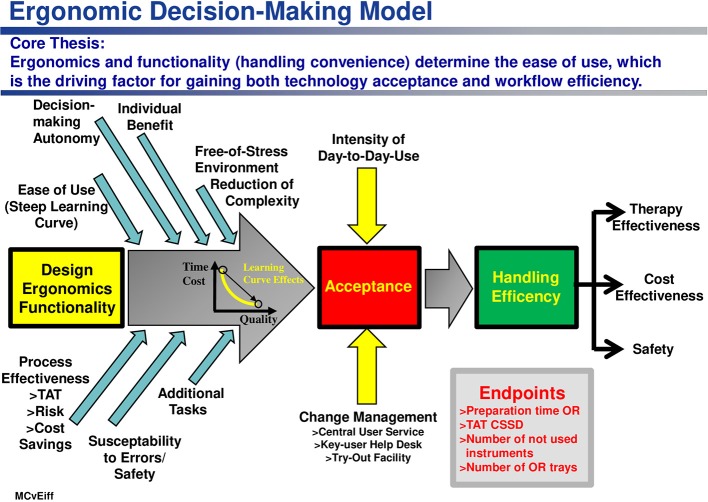
Ergonomics and functionality of a medical product (here: an instrumentation set) is a driving factor fostering acceptance of use and handling efficiency as a source of medical and economic effectiveness, as well as of patient safety.

From all these preliminary considerations, an ergonomic decision-making model (46) can be derived (see Figure 9) which establishes a relationship between

- The functionality of the OR trays used,
- The intervention-specific framework conditions within the OR,
- The satisfaction with the tray configuration by OR personnel on the one hand, and the
- Working efficiency as well as the
- Outcome variables of an OR process (process effectiveness, theater blocking time, cost effectiveness, degree of dissatisfaction on the part of the OR personnel, patient safety), on the other hand.

## Conclusion

The design, ergonomics and functionality of innovative medical products have a tremendous impact via the ease of handling, on costs, therapy effective-ness, work environmental and patient safety.

Given that ease of handling directly influences employee acceptance of a re-engineered process or an innovative medical product or disruptive technology, this can be regarded as a crucial precondition for the efficiency and effectiveness of workflows.

## Limitations of this Study

One limitation of the analysis is that clear recommendations pertaining successful change- management strategies and effective change management tools cannot be derived so far in this context. Further research and practical tests of selected organization-development interventions are needed.

A second limitation is that learning-curve effects could not identified and measured, due to a lack of robust criteria. Thus, the “time saved” in the sub-process of instrumentation logistics was used as a proxy for deriving information about process efficiency.

Thirdly, the analysis of opportunity costs is based on the premise that the department is working at full capacity. Yet, due to a lack of qualified staff in the German health care system, the number of hospitals working at only 70–75% capacity utilization is rising.

Furthermore, additional research aiming to identify differences in patient outcome in changing technology settings, should be intensified.

## Data Availability Statement

The datasets generated are not available for third parties due to data security requirements of staff-related data. Requests to access the datasets should be directed to WE, von.eiff@uni-muenster.de.

## Author Contributions

ME, WE, and MG developed the study design. ME designed the questionnaires, prepared the data analysis, wrote the first draft of the manuscript, integrated proposed revisions, and performed the manuscript revision. AR, WE, and MG wrote sections of the manuscript. AR and MG supported the data collection and obtained the permission of the work counsel. All authors read and approved the submitted version.

### Conflict of Interest

The authors declare that the research was conducted in the absence of any commercial or financial relationships that could be construed as a potential conflict of interest.
